# Paradoxical Interventricular Septal Motion as a Major Determinant of Late Gadolinium Enhancement in Ventricular Insertion Points in Pulmonary Hypertension

**DOI:** 10.1371/journal.pone.0066724

**Published:** 2013-06-24

**Authors:** Takahiro Sato, Ichizo Tsujino, Hiroshi Ohira, Noriko Oyama-Manabe, Yoichi M. Ito, Teruo Noguchi, Asuka Yamada, Daisuke Ikeda, Taku Watanabe, Masaharu Nishimura

**Affiliations:** 1 First Department of Medicine, Hokkaido University Hospital, Sapporo, Hokkaido, Japan; 2 Department of Diagnostic and Interventional Radiology, Hokkaido University Hospital, Sapporo, Hokkaido, Japan; 3 Department of Biostatistics, Hokkaido University Graduate School of Medicine, Sapporo, Hokkaido, Japan; 4 Department of Cardiovascular Medicine, National Cerebral and Cardiovascular Center, Suita, Osaka, Japan; 5 First Department of Medicine, Hokkaido University Graduate School of Medicine, Sapporo, Hokkaido, Japan; University of Giessen Lung Center, Germany

## Abstract

**Background:**

This study investigated the major clinical determinants of late gadolinium enhancement (LGE) at ventricular insertion points (VIPs) commonly seen in patients with pulmonary hypertension (PH).

**Methods:**

Forty-six consecutive PH patients (mean pulmonary artery pressure ≥25 mmHg at rest) and 21 matched controls were examined. Right ventricular (RV) morphology, function and LGE mass volume at VIPs were assessed by cardiac magnetic resonance (CMR). Radial motion of the left ventricular (LV) wall and interventricular septum (IVS) was assessed by speckle-tracking echocardiography. Paradoxical IVS motion index was then calculated. Univariate and multivariate regression analysis were conducted to characterize the relationship between LGE volume at VIPs and PH-related clinical indices, including the paradoxical IVS motion index.

**Results:**

Mean pulmonary arterial pressure (MPAP) of PH patients was 38±9 mmHg. LGE at VIPs was observed in 42 of 46 PH patients, and the LGE volume was 2.02 mL (0.47–2.99 mL). Significant correlations with LGE volume at VIPs were observed for MPAP (r = 0.50) and CMR-derived parameters [RV mass index (r = 0.53), RV end-diastolic volume index (r = 0.53), RV ejection fraction (r = −0.56), and paradoxical IVS motion index (r = 0.77)]. In multiple regression analysis, paradoxical IVS motion index alone significantly predicted LGE volume at VIPs (p<0.001).

**Conclusions:**

LGE at VIPs seen in patients with PH appears to reflect altered IVS motion rather than elevated RV pressure or remodeling. Long-term studies would be of benefit to characterize the clinical relevance of LGE at VIPs.

## Introduction

Pulmonary hypertension (PH) is defined as mean pulmonary arterial pressure (PAP) ≥25 mmHg at rest. [1] If untreated, the vasculopathy often progresses and leads to right ventricular (RV) failure and premature death. Optimal assessment of RV morphology and function is thus critical in the management of PH.

Cardiac magnetic resonance (CMR) imaging is an established modality for the objective assessment of RV geometry and function. To date, CMR studies of PH have described increased size and impaired function of the right ventricle. [2] Recent CMR studies have also demonstrated late gadolinium enhancement (LGE) at ventricular insertion points (VIPs). [3]–[10] LGE is commonly seen in patients with coronary and myocardial diseases and is thought to emerge as a result of contrast leakage and pooling in fibrotic or severely damaged myocardium. [11], [12] In PH, however, the underlying mechanisms of LGE at VIPs are insufficiently understood.

Regarding this, a possible explanation is that LGE at VIPs develops as a result of elevated RV pressure and resultant RV remodeling in PH. Indeed, previous studies have shown significant associations between LGE volume at VIPs and measurements of pulmonary hemodynamics and RV morphology. [4]–[6], [10] Interestingly, however, we have recently experienced and reported a case in which paradoxical motion of the interventricular septum (IVS) alone caused LGE at VIPs without PH. [13] This paradoxical IVS movement has been described in PH[14]–[16] and, thus, it can be assumed that altered IVS motion might be the predominant mechanism of LGE at IVS rather than increased RV pressure and/or remodeling.

The present study investigated the underlying mechanisms of LGE at IVSs in PH by evaluating the association between LGE at VIPs and clinical parameters. Particular focus was made on the possible contribution of paradoxical IVS motion assessed by speckle-tracking echocardiography to the development of LGE at IVSs.

## Methods

In this single-center, case-control, prospective, observational study, subjects who met the entry criteria [mean PAP of ≥25 mmHg and pulmonary capillary wedge pressure (PCWP) of ≤15 mmHg at rest] were consecutively enrolled between April 2010 and December 2012. Exclusion criteria were the presence of comorbid disease that might affect cardiac morphology and function, an unstable PH condition requiring treatment modifications, and inability to obtain or analyze electrocardiogram-gated CMR images. Patients with atrial fibrillation/flutter were excluded based on the last criterion. Age- and gender-matched subjects who did not have cardiac and/or respiratory diseases were recruited from the (para)medical staff of our institution to serve as control subjects. Subjects with systemic hypertension were considered eligible in both groups when blood pressure was well controlled and when echocardiography exhibited no structural changes of the left ventricle and atrium.

Patients with PH underwentechocardiography, CMR and right heart catheterization (RHC) within a 1-week interval during which they were clinically stable. RHC was conducted according to the guidelines for the diagnosis and treatment of PH, [1] and PAP, PCWP, RV end-diastolic pressure (EDP), right atrial (RA) pressure, and cardiac output (CO) were measured. CO was measured by the thermodilution method, and the mean of at least three measurements was used as representative data.

All PH patients and control subjects gave informed written consent to participate, and the study protocol was approved by the ethics committee of the Hokkaido University Graduate School of Medicine.

### CMR Study Protocol

CMR studies were performed using a 1.5-Tesla Philips Achieva magnetic resonance imaging system (Philips Medical Systems, Best, The Netherlands) equipped with Master gradients (maximum gradient amplitude, 33 mT/m; maximum slew rate, 100 mT/m/ms). Imaging was performed with 10–15 sec breath-holding during expiration, using a vector-cardiographic method for electrocardiogram gating. From coronal localizing images that demonstrated the gross cardiac anatomy, an orthogonal stack of axial slices was planned to cover the heart from a level just below the diaphragm to the bronchial bifurcation, covering the heart in diastole. Axial slices were acquired using a steady-state free precession pulse sequence (repetition time = 2.8 ms, echo time = 1.4 ms, flip angle = 60, acquisition matrix = 192×256, field of view = 380 mm, slice thickness = 10 mm, 0 mm inter-slice gap, and 20 phases/cardiac cycle). A slice thickness of 10 mm was used to minimize the number of image acquisitions, and to reduce the number and duration of the breath-holding.

Obtained images were evaluated using commercially available software (Extended MR Work Space: ver. 2.6.3, Philips Medical Systems, Amsterdam, The Netherlands). RV and left ventricular (LV) endocardial borders of contiguous axial slices were manually traced and the obtained time-volume curves allowed for calculation of RV and LV end-diastolic volume (EDV) and end-systolic volumes (ESV), and ejection fraction (EF). Epicardial ventricular borders were also manually contoured for quantification of the volume of RV and LV walls. The IVS was regarded as a part of LV wall. RV and LV masses were calculated by multiplying each wall volume by 1.05 g/cm^3^. Ventricular volume and mass were indexed by body surface area.

In patients with PH, Gd-DTPA (0.1 mmol/kg, Magnevist; Berlex Laboratories, Wayne, NJ) was intravenously administered. Ten minutes after the injection, a breath-holding, inversion-recovery (IR)-prepared, three dimensional turbo field echo pulse sequence with electrocardiogram gating was performed to obtain a delayed-enhancement image with fat saturation of spectral presaturation with inversion recovery. The imaging parameters were as follows: slice thickness = 5 mm; FOV = 400 mm; matrix size = 157×256; TR/TE = 3.8 ms/1.2 ms; flip angle = 15°; and number of signal averages = 1. For each subject, the inversion time was adjusted to null the signal from the normal myocardium; the typical inversion time was 260 to 280 ms. Hyper-enhanced regions at the anterior and posterior VIPs were manually contoured on each short-axis slice using the same software, yielding total LGE volume. This procedure was conducted by an examiner who was not aware of the measurements of echocardiographic study.

### Echocardiography

Echocardiograms were obtained using Vivid q (GE Healthcare, Milwaukee, WI). LV end-diastolic and end-systolic dimensions were assessed from the parasternal long-axis view. The eccentricity index, an index of IVS displacement toward left ventricle, was obtained using the parasternal short-axis view at both end-systole and end-diastole. [17]

**Figure 1:**
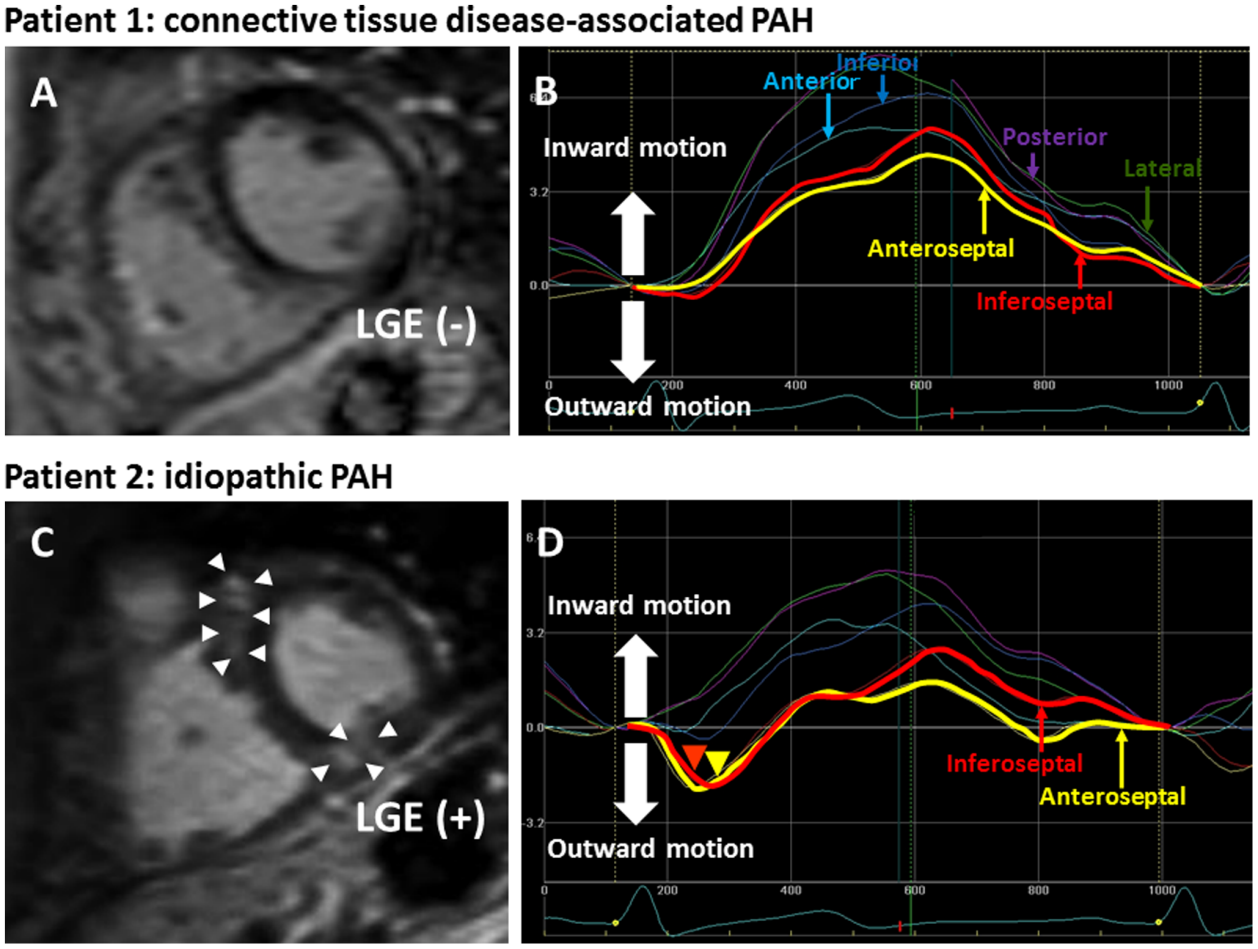
Representative images of cardiac magnetic resonance and speckle tracking echocardiography with or without late gadolinium enhancement and paradoxical motion of the interventricular septum. Patient 1 had connective tissue disease-associated PAH with a mean pulmonary artery pressure of 43 mmHg. CMR (A) shows no late gadolinium enhancement at ventricular insertion points. There is no paradoxical motion of the interventricular septum by speckle tracking echocardiography (B). Patient 2 had idiopathic PAH with a mean pulmonary artery pressure of 37 mmHg. Late gadolinium enhancement at ventricular insertion points is shown in a CMR image (arrow heads, C), and paradoxical motion of the interventricular septum at early systolic phase (arrow heads) is also noted on speckle tracking echocardiography (D). PAH, pulmonary artery hypertension; CMR, Cardiac magnetic resonance.

**Figure 2:**
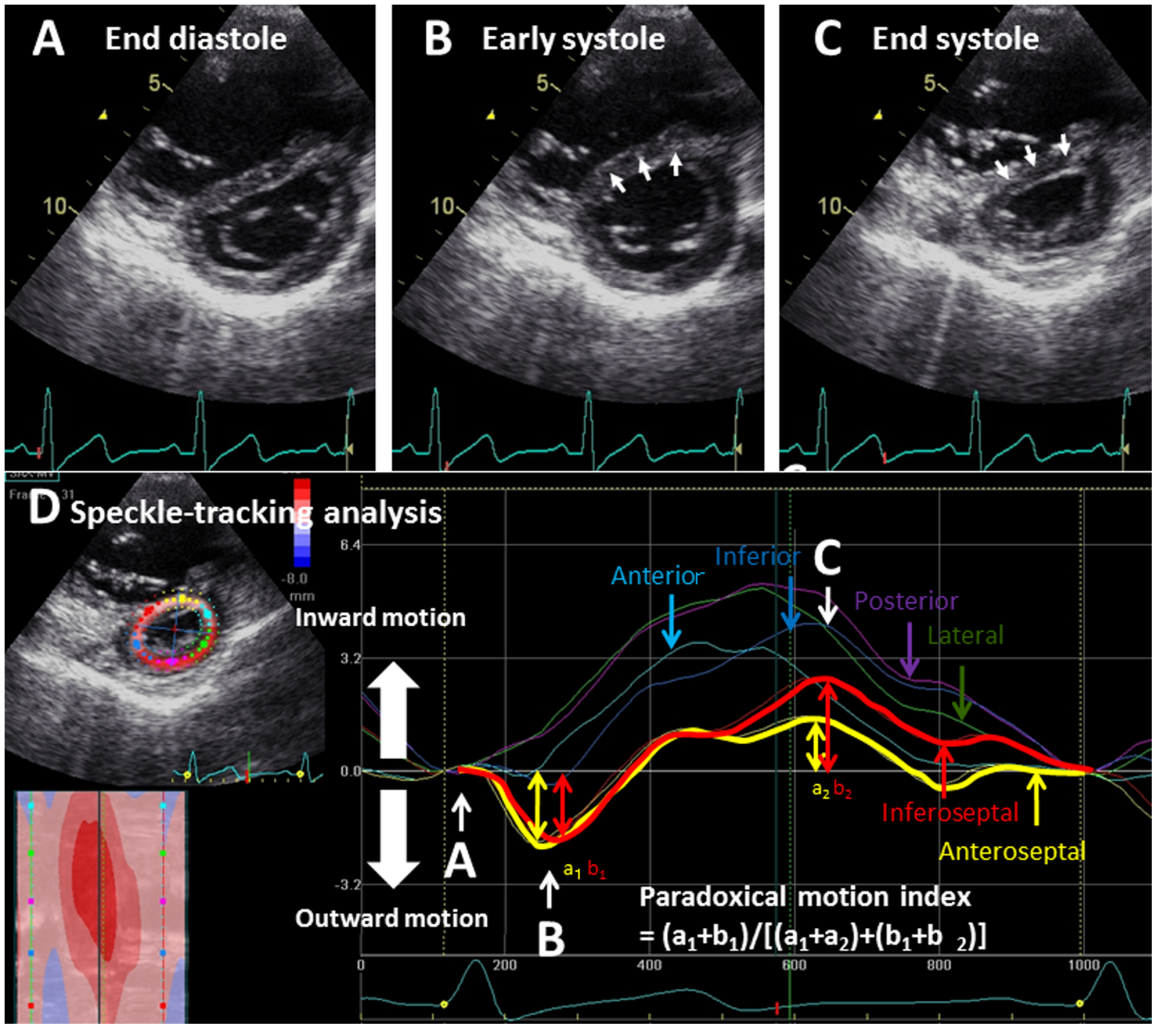
Quantitative assessment of paradoxical motion of the interventricular septum. Panels A, B and C show short axis images of the left ventricle at three cardiac phases of a patient with PH. From the end-diastolic phase (A) to the early systolic phase (B), the interventricular septum moves outward (toward right ventricle) in a paradoxical manner (B, arrows). Subsequently, from early systole (B) to end-systole (C), the interventricular septum moves inward in a manner similar to that of the other segments of the LV wall (panel C, arrows). However, the maximum inward motion was less than that of the other LV wall segments (D). To quantify the paradoxical interventricular septal motion indicated by arrows in panel B, we used speckle-tracking echocardiography by which the left ventricular free walls and interventricular septum were automatically divided into six segments, and each motion pattern was visualized by six lines (D). Yellow and red lines indicate the motion of the anterior and inferior interventricular septum, respectively, during a single cardiac cycle. The dips in the early systolic phase (a1 and b1) indicate their paradoxical motion. For quantitative assessment, the maximum depths of the yellow and red lines (a1+ b1) were added. Next, the entire distance of the two interventricular septal segments [early systole (a1 and b1) and end-systole (a2 and b2)] were added. Then, (a1+ b1) was divided by (a1+ a2+ b1+ b2), and the result was used as the paradoxical motion index of the interventricular septum.

For the objective assessment of the regional motion of the LV wall and IVS, established speckle-tracking analysis was conducted using commercially available software (EchoPAC, GE Vingmed, Horton, Norway). With this technique, LV free wall and IVS were automatically divided into six segments, and the radial motion of each segment was visualized by six lines in different colors. Figure 1A and 1B are representative images of a PH patient (patient 1) who showed no LGE at VIPs on a CMR image (A) and normal speckle tracking echocardiogram (B). Figure 2C is an image of another PH patient who showed LGE at VIPs on CMR. In the speckle tracking echocardiography of this patient, yellow and red lines are seen below the baseline in early systole (Figure 2D). Yellow and red lines represent the motion of the anterior and inferior IVS, respectively, indicating that the IVS moves paradoxically outward (toward right ventricle) in the early systolic phase in this case.

Figure 2 illustrates the manner of quantification of the paradoxical IVS motion. First, the paradoxical systolic motion of the anterior IVS (a1) and inferior IVS (b1) were added, and then the entire (inward and outward) motion of the anterior IVS (a1+ a2) and inferior IVS (b1+ b2) was calculated. Then, the summed paradoxical IVS motion (a1+ b1) was divided by the entire IVS motion [(a1+ a2)+(b1+ b2)], and the obtained quotient was used as the paradoxical IVS motion index.

### Reproducibility and Reliability of the Measurement of LGE Volume and Paradoxical IVS Motion Index

Intraobserver agreement for the two measurements: IVS volume and paradoxical IVS motion index was assessed by comparing the measurements of repeated analysis in five randomly chosen control subjects and in 10 randomly chosen PH patients (T.S.). Interobserver agreement of the two measurements was assessed using the same patients (n = 15) by comparing the results measured by T.S. and those obtained by a second, experienced cardiologist (I.T.). The second cardiologist was not aware of the echocardiographic measurements by the first examiner or of the CMR measurements. Bland-Altman analysis and intraclass correlations (ICC) were used to assess reproducibility.

### Statistical Analysis

Continuous variables are expressed as mean ± standard deviation (SD) for those normally distributed or otherwise as medians and interquartile ranges (IQR). Departures from normality were detected with the Shapiro-Wilk statistic. Differences in measurements between the control and PH groups were assessed with the chi-square test, Student’s *t* test, or the Wilcoxon test as appropriate. LGE volume in VIPs was compared between PH patients who were on vasodilation therapy and those who were not, and among PH patients treated with different regimens of vasodilators.

Correlations between LGE volume in VIPs and other clinical parameters were evaluated by Pearson’s correlation coefficient. The statistical power of our study was greater than 0.8 with a significance level of 0.05, [18] given the estimation based on the previous study that the correlation coefficients between LGE mass in VIPs and morphological and hemodynamic parameters of PH were 0.4 or greater. [4]

Multiple regression analysis was also conducted to examine if any measurement predicted LGE volume at VIPs in an independent manner. In this analysis, six variables that were likely to be associated with LGE at VIPs (i.e., mean PAP, RV EDV index, RV mass index, RV EF, diastolic eccentricity index, and paradoxical IVS motion index) were chosen as explanatory variables.

All statistical analyses were performed using JMP® Version 9 (SAS Institute Inc., Cary, NC), and p values less than 0.05 were considered to represent statistical significance.

## Results

A total of 51 Japanese patients met the entry criteria, but five patients were excluded based on pre-specified exclusion criteria. The mean duration between the initial recognition of any PH-related symptoms/signs and the diagnosis of PH was 43 months (range, 8–64). Twenty-one age- and gender-matched healthy controls were also enrolled. When comparing the 46 PH patients (male/female, 11/35; age, 50±15 years) and the 21 control subjects (male/female, 7/14; age 44±7 years), no significant demographic differences were apparent including in body mass index, smoking history and the prevalence of cardiovascular/respiratory diseases. Table 1 shows the characteristics of the PH patients.

**Table 1:** Characteristics of patients with pulmonary hypertension.

Number of patients		46
Diagnosis		
Pulmonary arterial hypertension/pulmonary veno-occlusive disease		23/1 (50/2%)
Pulmonary hypertension due to left heart disease		0 (0%)
Pulmonary hypertension due to lung diseases and/or hypoxia		6 (13%)
Chronic thromboembolic pulmonary hypertension		14 (30%)
Other		2 (4%)
**World health organization -functional class**		
II	22 (48%)	
III	20 (46%)	
IV	4 (7%)	
**Use of pulmonary hypertension-specific vasodilators**		
Beraprost	17 (37%)	
Sildenafil/tadalafil	11/1 (24%/2%)	
Bosentan/ambrisentan	14/1 (30%/2%)	
Intravenous epoprostenol	4 (9%)	
Combination therapy	17 (37%)	
None	19 (41%)	
**Pulmonary hemodynamics**		
Systolic pulmonary artery pressure (mmHg)	57 (46–75)	
Diastolic pulmonary artery pressure (mmHg)	24 (18–28)	
Mean pulmonary artery pressure (mmHg)	39 (30–43)	
Pulmonary capillary wedge pressure (mmHg)	8±2	
Right ventricular end-diastolic pressure (mmHg)	8±3	
Mean right atrial pressure (mmHg)	6±2	
Cardiac index (L/min/m^2^)	2.6 (2.4–3.1)	
Pulmonary vascular resistance (dyne⋅s⋅cm^−5^)	513 (386–785)	

Mean ± standard deviation for those normally distributed or medians and interquartile ranges.

### CMR Measurements in Controls and PH Patients

CMR images were acquired from all participants, and the image quality was sufficient for the subsequent analysis. When compared with control subjects, PH patients exhibited a greater RV EDV index (PH 98 [82–134] mL/m^2^, control 66 [52–77] mL/m^2^; p<0.001) and RV mass index (PH 36 [28–48] g/m^2^, control 19 [17]–[22] g/m^2^; p<0.001), and a smaller RV EF (PH 40 [32–47]%, control 52 [47–54]%; p<0.001) and LV EF (PH 59 [54–67]%, control 63 [61–69]%; p = 0.036). LGE at VIPs was found in 41 of 46 PH patients. Median LGE volume of PH patients was 2.02 [0.47–2.99] mL. LGE volume in VIPs of PH patients who were receiving vasodilators was 2.19 [0.49–3.02] mL, which was not significantly different from that of PH patients who were not on such treatment (1.83 [0.6–3.28] mL, p = 0.804). Also, no significant difference in LGE volume at VIPs were observed when comparing among the three different treatment regimens used in PH patients (p = 0.976) (beraprost alone, 0.65 [0.18–4.23] mL (n = 5); phosphodiesterase 5 inhibitor with or without beraprost, 2.27 [0.34–2.75] mL (n = 5); endothelin receptor antagonists with or without beraprost, 2.33 [1.19–2.44] mL (n = 9).

### Echocardiography Measurements in Controls and PH Patients

Echocardiography was completed in all subjects, and the image quality obtained was sufficient for all image analysis. The end-diastolic and end-systolic eccentricity indices were significantly greater in PH patients (end-diastole: 1.27 [1.10–1.39], end-systole: 1.45 [1.30 - 1.73]) than in controls (end-diastole: 1.00 [1.00–1.00], end-systole: 1.00 [1.00–1.01]) (p<0.001 for both indices). Paradoxical IVS motion index was also significantly greater in PH patients (0.23±0.14) than in controls (0.04±0.03) (p<0.001).

### Associations of LGE Volume at VIPs with Clinical Parameters of PH

LGE volume at VIPs significantly correlated with mean PAP (r = 0.50, p<0.001), RV EDV index (r = 0.53, p<0.001), RV ESV index (r = 0.59, p<0.0001), RV mass index (r = 0.53, p<0.001), RV EF (r = −0.56, p<0.001), end-diastolic eccentricity index (r = 0.60, p<0.001), end-systolic eccentricity index (r = 0.55, p<0.001), and paradoxical IVS motion index (r = 0.77, p<0.001), but not with cardiac index (CI) (r = −0.06, p = 0.657), pulmonary vascular resistance (PVR) (r = 0.28, p = 0.06), LV EDV index (r = −0.07, p = 0.63), LV ESV index (r = 0.09, p = 0.56), LV mass index (r = 0.10, p = 0.543), LV EF (r = −0.26, p = 0.078), and the disease duration of PH (r = 0.17, p = 0.25).

**Figure 3:**
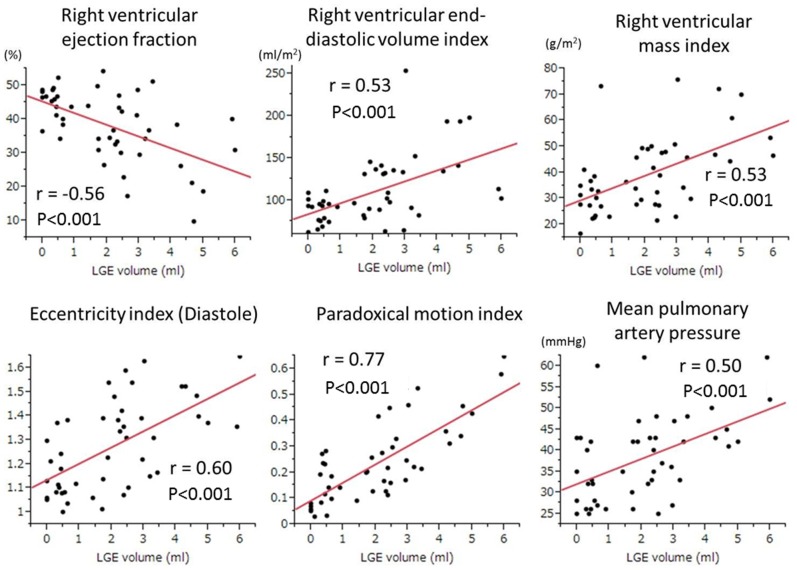
Relationships between late gadolinium enhancement volume at ventricular insertion points and other clinical parameters. Among the six clinical indices of pulmonary hypertension, the paradoxical motion index of the interventricular septum exhibited the highest correlation coefficient (r = 0.77, p<0.001) with late gadolinium enhancement volume.

Figure 3 shows associations between LGE volume at VIPs and six prespecified explanatory variables chosen for multivariate analysis. Among these six variables, paradoxical IVS motion index indicated the highest regression coefficient (6.27). Also, in the multiple regression analysis, paradoxical IVS motion index significantly predicted LGE volume at VIPs (p<0.001) but other five variables did not **(**mean PAP, p = 0.463; RV EDV index, p = 0.64; RV mass index, p = 0.933; RV EF, p = 0.264; and diastolic eccentricity index, p = 0.626).

### Reproducibility and Reliability of the Measurements of LGE Volume and Paradoxical IVS Motion Index

Bland-Altman analysis of the intraobserver variability of LGE volume at VIPs showed low mean differences and limits of agreement (0.02±0.30 ml). The ICC was 0.99. Regarding interobserver variability, Bland-Altman analysis showed similarly small mean differences and limits (0.2±6.1 ml). The ICC was 0.97.

Bland-Altman analysis of the intraobserver variability of the paradoxical IVS motion index showed low mean differences and limits of agreement (0.8% ±3.5%). The ICC was 0.98. Regarding interobserver variability, Bland-Altman analysis revealed similarly small mean differences and limits (−1.0% ±4.4%). The ICC was 0.96.

## Discussion

In the present prospective observational case-control study, 46 PH patients and 21 matched controls were examined. Results suggested the following: (1) LGE at VIPs is a common CMR finding (positive in 42 (91%) of 46 PH patients); (2) early systolic paradoxical motion of VIPs is also common; (3) the volume of LGE correlates with the pulmonary hemodynamics, and with CMR and echocardiographic parameters of the right ventricle; and (4) the paradoxical IVS motion index is the only independent explanatory variable of LGE volume at VIPs in PH.

Recent studies have indicated significant associations between LGE at VIPs and various parameters of PH. For example, mean PAP, RV volume and RV mass index have been reported to positively correlate with LGE volume. [4], [6], [10] Also, functional parameters such as RV EF and CI have also been shown to inversely correlate with LGE volume at VIPs. [4]–[6], [10] Indeed, the results of univariate analysis in the present study were consistent with observations from these previous publications. Importantly, however, no prior studies have examined the possible link between LGE at VIPs and the pattern of IVS motion during a cardiac cycle. In the present study, we indexed the degree of paradoxical IVS motion using speckle tracking echocardiography and found that the degree of such IVS motion is an independent explanatory variable of LGE at VIPs in PH. Conversely, MPAP and other RV indices did not predict LGE volume at VIPs in multivariate regression analysis.

Abnormal IVS motion has been documented by M-mode [14], [15] and by speckle tracking echocardiography in PH. [19] Previous reports have focused on the underlying mechanism or the impact of this IVS motion on the overall cardiac performance. In line with these reports, the present study demonstrated significant associations of the paradoxical IVS motion index with the pulmonary hemodynamic measurements and RV EF. However, the main focus of the present study was the possible regional impact of paradoxical IVS motion on VIPs. Regarding this issue, two prior animal studies (dog PH model) demonstrated that myocardial tissue at VIPs is prone to encounter pull and increased tension. [20], [21] Also, Spottiswoode et al. reported that paradoxical IVS motion can generate high stresses and strains at VIPs in a non-PH patient. [22] These prior publications, along with the results of the present study, suggest that LGE at VIPs might develop due to the mechanical impact of paradoxical IVS motion on VIPs irrespective of PH.

Contrast pooling at VIPs was suggested as the primary mechanism of LGE in PH in a recent autopsy report. [8] Notably, this report demonstrated disarrayed myocardium but no abnormal fibrosis or damaged myocardium at VIPs. Accordingly, the authors speculated that contrast pooling in the widened intermyocardial fibers caused LGE at VIPs in their case. The present study partly supports this notion, because paradoxical IVS motion is likely to affect the architecture of the myocardial tissue at VIPs, as was reported in prior studies. [20], [22]

Freed et al. recently reported that the presence of myocardial LGE at VIPs is a marker of poor prognosis in PH [9]. In this regard, the present study showed that LGE at VIPs was independently associated with paradoxical IVS motion but not with established predictors such as RV mass and EF [23]. Indeed, LGE at VIPs may be a sole reflection of paradoxical IVS motion and resultant contrast pooling [8] and thus whether LGE at VIPs can be a better prognostic marker over previously reported indices needs to be investigated in future prospective studies.

One limitation of the present study is the inclusion of PH patients with diverse etiologies. Some underlying diseases of PH are known to affect the myocardium; thus, the experimental results might have been affected by the different underlying illnesses among the patient population. Also, in the subgroup analysis, the number of PH patients on different treatment regimens was small. Thus, any confounding influence of the specific treatment on the experimental results cannot be excluded. Also, the present study analyzed patients with relatively less advanced PH, which makes it difficult to extrapolate the findings of this study to those with advanced PH. Further, IVS motion was evaluated using a methodology that is not commonly used. However, speckle-tracking echocardiography is an established method of regional analysis of IVS motion. Indeed, the calculated intra- and inter-observer reproducibility of the paradoxical IVS motion index were favorably high. Lastly, we did not conduct pathological observations in the present study. Thus, further clinicopathological studies are warranted to clarify the true mechanisms of LGE at VIPs in PH.

In conclusion, the present CMR study of PH demonstrated an independent association of LGE at VIPs with early systolic paradoxical IVS motion, but not with indices of pulmonary hemodynamics and RV morphology. This suggests that LGE at VIPs is a hallmark of altered IVS motion but not of PH per se. The clinical relevance of this distinct CMR finding must be clarified in future long-term studies.
